# CBCT-Aided Microscopic and Ultrasonic Treatment for Upper or Middle Thirds Calcified Root Canals

**DOI:** 10.1155/2016/4793146

**Published:** 2016-07-25

**Authors:** Ying-Ming Yang, Bin Guo, Li-Yang Guo, Yan Yang, Xiao Hong, Hong-Ying Pan, Wen-Ling Zou, Tao Hu

**Affiliations:** ^1^State Key Laboratory of Oral Diseases, West China College of Stomatology, Sichuan University, Chengdu, Sichuan 610041, China; ^2^Institute of Stomatology, Chinese PLA General Hospital, Beijing 100081, China

## Abstract

Root canal calcification is considered a great challenge during root canal treatment. Although the application of ultrasonic instruments and dental operating microscope (DOM) has advantages, dealing with calcified root canals still suffers a great risk of failure because of limited information about the location, length, and direction of obliteration on periapical radiographs. In this work, a cone-beam computed tomography- (CBCT-) aided method aimed at solving complicated calcified root canals in which conventional approaches could not work was proposed. Thirteen teeth with sixteen calcified canals (12 calcified in the upper third, 4 calcified in the middle third), which cannot be negotiated with conventional methods, were treated with the aid of CBCT. The location of calcification and depth of instrumentation and operating direction were calculated and assessed in three dimensions with ultrasonic instruments under DOM. In all thirteen teeth, canals with upper and middle thirds calcification were treated successfully. Finally, a guideline was proposed to help achieve consistent apical patency in calcified canals.

## 1. Introduction

Root canal treatment (RCT) is generally considered to be the most effective treatment for endodontic diseases including irreversible pulpitis and apical periodontitis [1, 2]. Effective control of the resident microbiota in pulp and periapical tissue includes proper shaping, disinfection, and obturation of the root canal system. Whether instrumentation can access the full length of root canals or not may greatly affect the prognosis [3, 4]. But a variety of factors may influence the outcomes of root canal negotiation and penetration, such as curvature, obliteration, and transportation [5, 6]. Root canal calcification, which refers to the deposition of hard tissue on the root canal wall as a result of trauma, caries, periodontal diseases, and aging, contributes to 76.7% of difficult RCT cases [7, 8].

At present, the main methods to deal with root canal calcification can be summarized as follows: (a) gradual instrumentation and cleaning using a small size of K-files or C+ files with ethylenediaminetetraacetic acid (EDTA) [9] and (b) removal of calcification with ultrasonic equipment under dental operating microscope (DOM) [10, 11]. However, the rate of treatment failure for calcified canals has been reported to range from 20% to 70% and it depends on the dentists' clinical experience and knowledge of anatomy and X-ray examinations [7]. Even aided with the combination of DOM and ultrasonic instruments, only 74.0% success rate can be reached [8].

X-ray radiographs, which are conventionally used in endodontic treatment following ALARA-principles, are revealed to be defective because of shadows, anatomical noise, geographical distortion, overlapping, elongation or depression of root canals, and so forth [12]. Buchanan pointed out that almost all the root canals had certain degrees of curvature, especially existing in the buccolingual direction situation, and it could not be fully demonstrated on an ordinary X-ray film [13]. In this context, the access to calcified teeth has to face a high risk of unpredictable dentin destruction in the buccolingual direction. The calcification of root canal orifices can render locating the root canal more difficult. Although such calcification is usually distinguished from dentin in colour, the differentiation is still confusing for the dentists with less clinical experience. In the case of a slight difference, even experienced dentists can be frustrated.

A contemporary development of oral imageology, cone-beam computed tomography (CBCT), obtains volumetric information from continuous cross-sectional scanning with spiral CT, and these data can be used in three-dimensional reconstruction [14]. The image overlapping and distortion frequently encountered on routine X-ray films can be obviously reduced. CBCT provides not only three-dimensional views but also morphologic details of the root canal system, which were considered superior to routine X-ray films [15, 16]. However, the analysis of CBCT data in calcified teeth seems variable which depends on the dentists' clinical experience, leaving a dubious perspective of the application of this technique.

The aim of the present study was to assess the treatment effects of CBCT-aided RCT in root canals with upper or middle thirds calcification that cannot be accessed via traditional endodontic therapy. Finally, a sequential protocol was proposed to guide the clinicians when negotiating root canal calcification.

## 2. Methods

### 2.1. Patients

We recruited patients at the Department of Endodontics, West China Hospital of Stomatology, Sichuan University, from March 2012 to April 2014. The participants received RCT or endodontic retreatment with root canals calcified in upper or middle thirds. Firstly, size 8 and 10 K-files were handled with a gentle watch-winding motion with minimal vertical pressure with ethylenediaminetetraacetic acid (EDTA) to negotiate the calcified root canals [17]. Once this succeeded, the case was excluded. Otherwise, the patients would receive further endodontic therapy under the DOM with ultrasonic instruments. Those cases that still failed were final subjects eligible for the present study. Three endodontic specialists accomplished all cases and each case was in the charge of the same endodontist. The IRB approved the protocol and informed consent was obtained from each patient. Thirteen teeth with 16 root canals were included (Table 1).

### 2.2. CBCT Scanning

An experienced operator acquired the CBCT images using 3D Accuitomo Tomograph (Morita, Kyoto, Japan). The operating parameters were 80 kV, 5.0 mA, and an exposure time of 17.5 s. The voxel size was 0.125 mm and the slice thickness was 1.0 mm. Scanning was conducted according to the manufacturer's recommended protocol.

### 2.3. CBCT Analysis

The following analysis was accomplished with computer software.

*(1) Determination of Root Canal Curvature*. Three-dimensional images could assist in calculation of the degree, diameter, and length of root canal curvature [18], which helped evaluate the level of difficulty of RCT and guide the treatment procedure.

**Figure 1:**
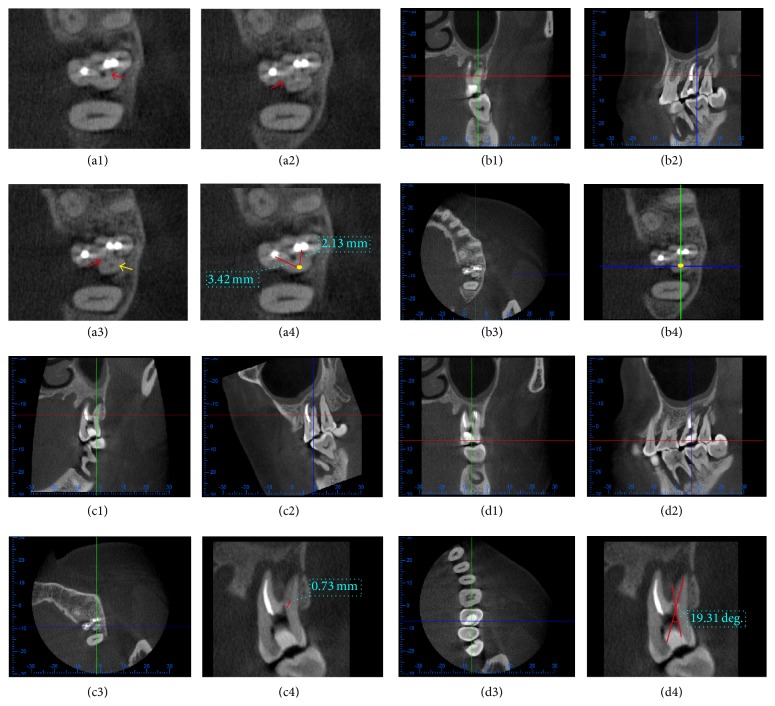
Determination of Parameters. (a1)–(a4) Locate calcified root canal orifice. On cross-sectional plane, canal managed by ultrasonic instrument before CBCT scanning (red arrows) and real canal below calcified orifice (yellow arrow) could be visible. Then, the distance between the calcified orifice and other references (usually orifices of other canals) could be measured to help locate the calcified orifice. (b1)–(b4) Adjust the cross-sectional plane, by moving the red line, to make clear root canal image under the calcification visible. Then, move blue line and green line until their intersection locates on the canal under the calcification, which made the canal appear on buccolingual and mesiodistal planes. (c1)–(c4) Determine the depth of calcification. Rotate the mesiodistal plane to make the blue line in accordance with the same trend of the root canal and overlap the canal resulting in the maximum exposure of root canal on buccolingual plane. Then, the depth of calcification (DOC) in buccolingual direction was determined. In the same way, DOC in mesiodistal direction could be measured (not shown). (d1)–(d4) Determine the direction of calcification. On cross-sectional plane, move the blue line parallel to the mesial or distal wall of pulp chamber (distal wall in this figure) to make the measurement plane in buccolingual direction parallel to distal wall of pulp chamber. Then, the buccolingual instrument angle (BLIA) was determined. In the same way, mesiodistal instrument angle (MDIA) could be measured (not shown).

*(2) Determination of the Depth of Calcification and Instrumentation*. The cross-sectional plane was adjusted by moving the red line on mesiodistal/buccolingual planes until the clear root canal image beneath the calcification appeared and the mesiodistal/buccolingual planes were rotated to the same trend of the root canal, which resulted in the maximum exposure of root canals in mesiodistal and buccolingual planes, respectively (Figure 1(b)). Then, the depth of calcification (DOC) was determined in mesiodistal and buccolingual directions using analysis software (Figure 1(c)).

*(3) Determination of the Location of the Calcified Root Canal Orifice (Figure 1(a))*. The red line was adjusted to the lower border of the pulp chamber in the buccolingual or mesiodistal plane following step (2), and the three planes would intersect at this point—the calcified orifice of root canal. On the cross-sectional plane, the intersection between the blue line (representing buccolingual plane) and green lines (representing mesiodistal plane) was determined as the calcified orifice of the root canal. The distance between the root canal orifice and its references (usually orifices of other canals) could be measured, which could help locate the calcified root canal orifice.

*(4) Determination of the Instrumentation Angle for Calcified Canals (Figure 1(d))*. The buccolingual plane (blue line) was adjusted to parallel the mesial or distal wall of pulp chamber on the cross-sectional plane. In this way, the extension of the line of canal calcification and the buccal wall of the pulp chamber formed an angle on the buccolingual plane, which we determined as the buccolingual instrumentation angle (BLIA). In the same way, the mesiodistal plane was adjusted according to the buccal or lingual wall of pulp chamber, and the mesiodistal instrumentation angle (MDIA) could be defined.

All the measurements were conducted in triplicate and the average values were calculated in each step.

### 2.4. Root Canal Negotiation and Penetration

With three-dimensional information of the location, depth, and direction of the calcification, access to the calcified teeth under DOM with ultrasonic instruments was accomplished by the same endodontic specialist at the first visit.

### 2.5. Statistics

The number of successful and failed root canals was recorded. Procedural success was defined as the achievement of apical patency without root perforation. Procedural failure was defined as (1) the occurrence of perforation or (2) inability to recanalize the root canal after the ultrasonic instrument had been advanced to a position deemed at 0.5 mm below the lower end of the calcification based on CBCT.

## 3. Results

With the aid of CBCT, DOM, and ultrasonic instrument, all the 16 calcified canals (12 calcified in the upper third, 4 calcified in the middle third) were successfully negotiated. A 100% rate of success was achieved.

Representative cases are reported as follows.

### 3.1. Case  1: Calcification of the Root Canal Orifice (Figure 2)

A 19-year-old male patient had suffered from autonomous pain and radiation pain in the right posterior teeth region for 3 days before he consulted a dentist and was diagnosed with acute pulpitis on tooth 16. During conventional RCT, only the palatal canal was found. The patient was then referred to our department for further treatment.

The physical examinations demonstrated that the tooth had a huge coronal defect and the pulp chamber had already been exposed. There was no detectable mobility on tooth 16, with neither percussion nor cold/hot induced pain. Only mesiobuccal (MB) and palatal canals could be detected by size 8 and 10 K-files under DOM. The distobuccal canal still was not found even when the ultrasonic instruments were placed into the canal orifice at the depth of about 2 mm. After the patient's consent was obtained, a CBCT examination revealed that there were 4 canals including a distobuccal (DB) canal and the second mesiobuccal canal (MB2), both with calcified orifices. For instance, the mean length of DB canal calcification was 1.85 mm according to CBCT software analysis. The distance between the calcified orifices and related references (orifices of the palatal and MB canal) was measured, and calcified orifices were located. The BLIA was 23.35° and PDIA was 11.24°. Then, MB2 analysis was in the same way. After rubber dam isolation, the calcification was successfully removed with ultrasonic instrument along the calculated angle to reach the determinate depth. Then, the working length was determined by an electronic apex locator (Root ZX, Morita, Kyoto, Japan), and all canals were shaped with K3 Nickel titanium rotary equipment (SybronEndo, CA, USA) and filled with hot gutta-percha (SuperEndo*α*/*β*, CA, USA) and AH Plus (Dentsply, Konstanz, Germany) following the manufacturer's protocol.

### 3.2. Case  2: Root Canal Calcification in the Middle Third (Figure 3)

A 36-year-old male patient had suffered from autonomous pain and pain in occlusion in the left upper posterior teeth region for a month before consulting a doctor. After being diagnosed with chronic periapical periodontitis of tooth 27 and calcified root canals were determined, he was referred to our department. Clinical examinations revealed that tooth 27 was temporarily filled, with no mobility and no pain on percussion. There was a fistula in the buccal gingival tissue of the tooth. An X-ray image failed to reveal a clear view of the canal system, but periapical shadows and a depression-like resorption of the proximal alveolar bone were observed.

After the temporary filling was removed, only buccal canal was unobstructed while the palatal canal was only found to be open at the middle third level. After the failure of negotiating with ultrasonic instrument, a treatment plan was proposed including a CBCT examination, RCT with ultrasonic instrument under DOM, and basic periodontal treatment for the tooth. The patient consented to this treatment.

CBCT revealed that tooth 27 had 2 roots with single canal that could be defined as a type 3 variation for upper second permanent molars according to Zhang et al. [19]. In the middle third of the palatal canal, there was still calcification approximating to 3 mm in length. However, the apical third of the canal remained clear in the CBCT images. The curvature was 15° and this canal could be categorized with a medium level of difficulty according to the Schneider classification [20]. The BLIA was 15.24° and PDIA was 6.78°. After rubber dam isolation, the calcification was successfully removed with DOM and ultrasonic instrument; then preparation and obturation of root canals were accomplished.

### 3.3. Case  3: Deviation in Instrumentation Direction during RCT for Calcified Canal (Figure 4)

A 52-year-old female patient was referred to our department for obstruction of the distal buccal canal in another clinic. Tooth 27 was sealed with white temporal material. There was no mobility of the tooth and no pain on percussion. Periapical radiograph revealed that the mesiobuccal and palatal canals were obturated, but the distobuccal canal was not treated with a large periodontal transparent area. Three orifices except distobuccal canal were found to be exposed to the pulp chamber. The orifice of the distobuccal canal was located under DOM. After the ultrasonic instruments achieved at the depth of about 2 mm downward the canal orifice, the direction of canal pathway seemed to be lost. Then, we doubted if we had not gone toward the right direction.

Thus, a CBCT examination was performed after the patient's consent was obtained. The distobuccal canal was found calcified in the upper third, with a length of calcification of about 1 mm. The original path of instrumentation had obviously deviated in the mesiolingual direction. Subsequently, the BLIA and PDIA were determined using the method described above and the angle of instrumentation was accordingly adjusted distolingually. After the calcification was successfully removed with DOM and ultrasonic instrument, the root canal was prepared and obturated following working length determination.

## 4. Discussion

Although root canal therapy is approved to reveal predictable healing in a majority of cases, the prognosis is still uncertain in the cases of calcified teeth. Either persistent apical periodontitis or residual pulpitis is the well-known complication after a failed RCT of calcified root canals. Results of this pilot study showed the potential in the success rate increase of RCT regarding these complicated cases, and more attention and exploration still are needed in future investigations.

In recent years, CBCT has been proven to be useful for analyzing periapical diseases and the root canal system, which makes it more conventional in dental implantation, apical surgery, and complicated RCT [21–23]. It has also been used to measure the distance between different root canal orifices in mandibular first permanent molars [24]. In this study, the CBCT was revealed to take advantages of good resolution and three-dimensional presentation. With the aid of CBCT, adverse events such as perforation and deviation have been avoided in 16 root canals. Treatment for all of the selected clinical cases achieved a 100% success rate, which revealed a great improvement to deal with the treated calcified root canals after failed management through routine approaches.

In spite of the superiority of CBCT, CBCT is still not recommended instead of traditional X-ray films during initial steps in endodontic application under consideration of the ALARA-principles and ultrasonic instruments and DOM are not suggested as principal approaches. A guideline was proposed for teeth with obliterated root canal systems:

1. Firstly, size 8 and 10 K-files and C+ files were handled using a gentle watch-winding motion with minimal vertical pressure to negotiate the calcified root canals with ethylenediaminetetraacetic acid (EDTA) [17].
2. Once failed, the root canal therapy under the DOM with ultrasonic instruments was recommended. Success could be achieved in most cases except for those with calcified root canal orifices.
3. With a long calcification or a wrong instrumentation direction, the canal could be still blocked even if the depth of ultrasonic instruments exceeded 2 mm. Then, a CBCT scanning was recommended in teeth with upper or middle thirds calcification, followed by analysis of location, length, and direction of obliteration. And management under DOM with ultrasonic instruments could be conducted accordingly.

The analysis of CBCT data in calcified teeth not only provides a clear 3D depiction and detects root canal anatomy more accurately but also assists the dentists in operative design including selection of the optimum approach. Although access to the calcified teeth is important, avoiding complications of unexpected and excessive dentin destruction is also essential. The digital planning procedure can provide a directional 3D visualization and predictable prognosis to decrease the risk of complications before RCT. However, this method may leave the dentists with a mismatch between the planning and execution if the dentists do not have a good 3D mental visualization and a steady hand. Therefore, templates are printed to guide the clinician when locating the canal opening in anterior teeth with root canal calcification [25, 26]. But the volume and thickness of the templates restrict its application in posterior teeth. In the future, developments will focus on feasible and directional guide for locating obliterated canals in posterior teeth.

## 5. Conclusions

The presented sequential protocol as well as normative analysis of CBCT data seems to be a safe and clinically effective method for teeth with upper and middle thirds calcification. Digital design is a vital step for locating root canals and preventing root perforation. The application of CBCT changes empirical operation into quantitative operation while increasing the predictability of the complicated root canal treatment.

## Figures and Tables

**Figure 2:**
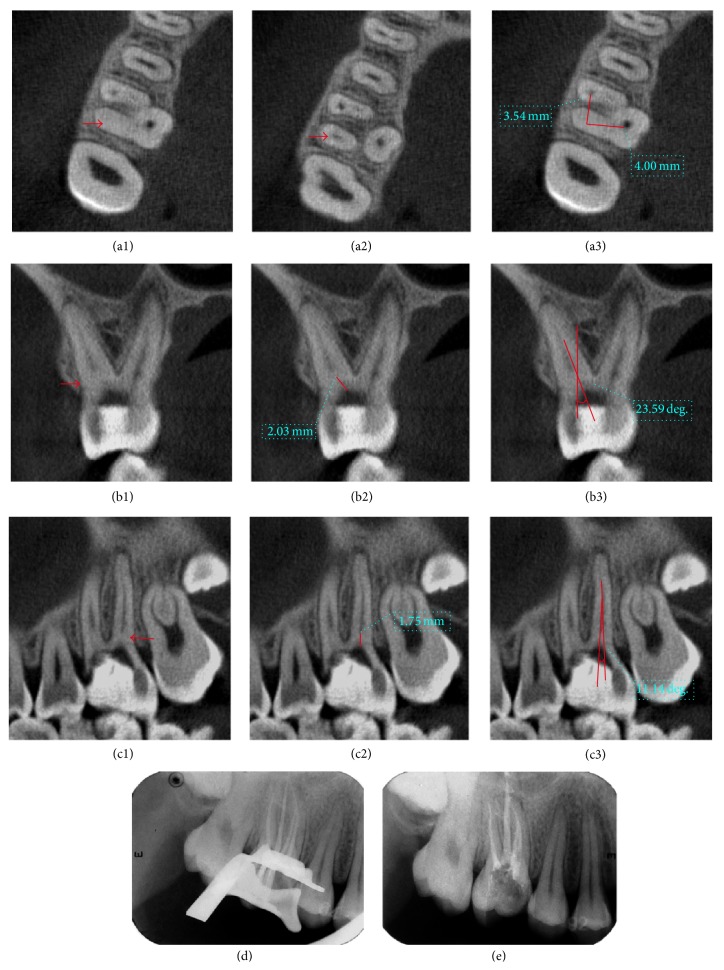
Case  1: (a1)–(a3) determination of the location of calcified root canal orifice: (a1) calcified orifices (red arrows); (a2) root canals under calcification (red arrows); (a3) and distance between the calcified orifice and orifices of other canals (red lines). (b1)–(b3) Determination of depth in buccolingual direction and BLIA. (c1)–(c3) Determination of depth in mesiodistal direction and MDIA. (d) Affirmation of working length. (e) Canals after filling and temporary restoration.

**Figure 3:**
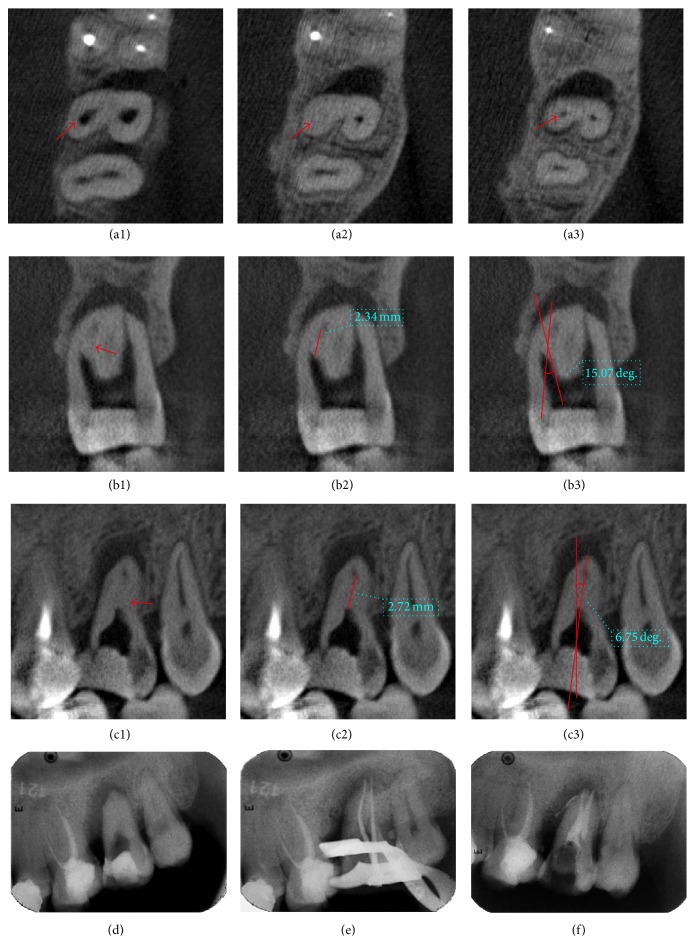
Case  2: (a1) upper part of the palatal canal over calcification managed by ultrasonic instrument before CBCT scanning (red arrow). (a2) Calcification of palatal canal (red arrow). (a3) Canal under calcification (red arrow). (b1)–(b3) Determination of depth in buccolingual direction and BLIA. (c1)–(c3) Determination of depth in mesiodistal direction and MDIA. (d) Radiograph before operation. (e) Affirmation of working length. (f) Canals after filling and temporary restoration.

**Figure 4:**
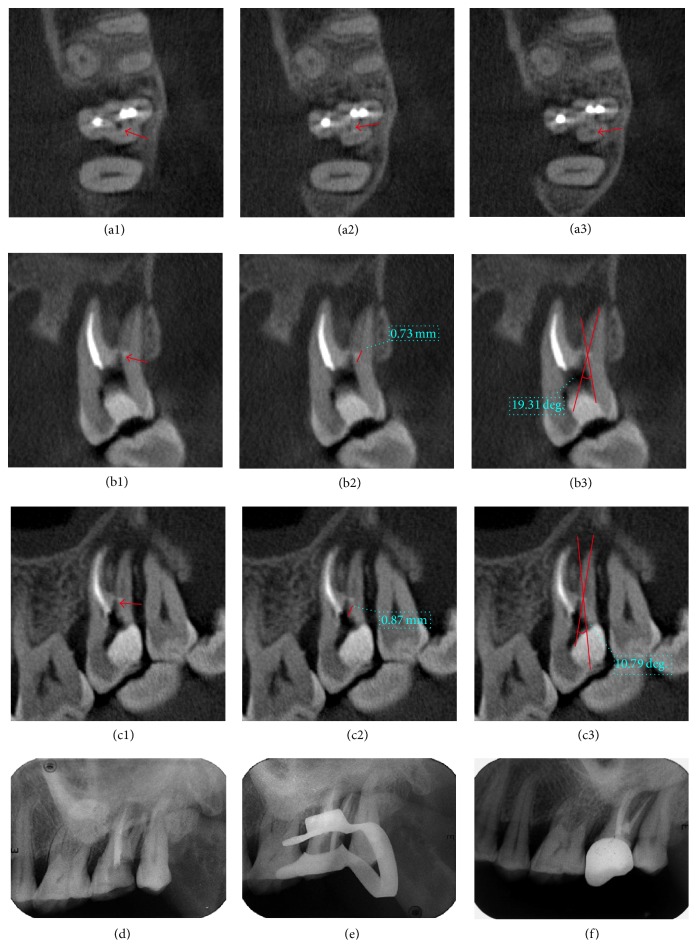
Case  3: (a1)–(a3) distobuccal canal managed by ultrasonic instrument before CBCT scanning (red arrow). (a2) Calcification of palatal canal (red arrow). (a3) Canal under calcification (red arrow). (b1)–(b3) Determination of depth in buccolingual direction and BLIA. (c1)–(c3) Determination of depth in mesiodistal direction and MDIA. (d) X-ray radiograph before operation. (e) Confirmation of working length. (f) Canals after filling and restoration.

**Table 1:** Summary of calcified root canals treated in the study.

Jaw	Tooth type	Number of teeth	Number of calcified root canals	Position of the calcification
Maxilla	First premolar	1	1	Middle 1/3
	First molar	3	5	Upper 1/3
		1	1	Middle 1/3
	Second molar	4	5	Upper 1/3
		1	1	Middle 1/3

Mandible	First molar	2	2	Upper 1/3
	Second molar	1	1	Middle 1/3

Total		13	16	
